# Gastroesophageal Reflux Disease and Probiotics: A Systematic Review

**DOI:** 10.3390/nu12010132

**Published:** 2020-01-02

**Authors:** Jing Cheng, Arthur C. Ouwehand

**Affiliations:** DuPont Nutrition & Biosciences, Global Health & Nutrition Science, Danisco Sweeteners Oy, Sokeritehtaantie 20, FI-02460 Kantvik, Finland; arthur.ouwehand@dupont.com

**Keywords:** gastroesophageal reflux disease, regurgitation, heartburn, probiotics

## Abstract

Probiotic is little known for its benefits on upper gastrointestinal health. The objective of this systematic review was to examine the efficacy of probiotics in alleviating the frequency and severity of symptoms in gastroesophageal reflux disease (GERD) in the general adult population. The PubMed and Web of Science databases were searched for prospective studies on GERD, heartburn, regurgitation, and dyspepsia, without any limitation on sample size. The Jadad scale was used to evaluate the quality of randomized controlled trials. In total, 13 prospective studies that were published in 12 articles were included in the analysis and scored per the Jadad scale as high- (five studies), medium- (two), and low- (six) quality. One article reported on two probiotic groups; thus, 14 comparisons were included in the selected studies, of which 11 (79%) reported positive benefits of probiotics on symptoms of GERD. Five out of 11 positive outcomes (45%) noted benefits on reflux symptoms: three noted reduced regurgitation; improvements in reflux or heartburn were seen in one study; five (45%) saw improvements in dyspepsia symptoms; and nine (81%) saw improvements in other upper gastrointestinal symptoms, such as nausea (three studies), abdominal pain (five), and gas-related symptoms (four), such as belching, gurgling, and burping. In conclusion, probiotic use can be beneficial for GERD symptoms, such as regurgitation and heartburn. However, proper placebo-controlled, randomized, and double-blinded clinical trials with a sufficient number of participants are warranted to confirm its efficacy in alleviating these symptoms. Further, interventions with longer durations and an intermediate analysis of endpoints should be considered to determine the proper therapeutic window.

## 1. Introduction

Gastroesophageal reflux disease (GERD) is a common digestive disorder in the general population that primarily affects the esophagus and gastro-duodenum. Due to its prevalence, GERD has a significant impact on quality of life (QoL) and healthcare costs.

### 1.1. Definition

The World Gastroenterology Organization defines GERD as ‘troublesome symptoms sufficient to impair an individual’s quality of life, or injury or complications that result from the retrograde flow of gastric contents into the esophagus, oropharynx, and/or respiratory tract’ [1]. The Rome IV criteria include functional heartburn (FH) and reflux hypersensitivity (RH), which can overlap with GERD [2]. Further, the Rome IV criteria describe infant regurgitation (IR) as follows: regurgitation 2 or more times per day for 3 or more weeks [3], spontaneous resolution with age, and no association with negative long-term consequences [4]. IR is not included in this systematic review.

Typical symptoms of GERD are heartburn and regurgitation, rendering the distinction between GERD, FH, and RH complicated. To improve the diagnosis of GERD, the Gastroesophageal Reflux Disease Working Group of the International Working Group for Gastrointestinal Motility and Function created a consensus document to determine modern indications for esophageal testing in GERD and define criteria for the clinical diagnosis of GERD [5]. Diagnosis and investigation of GERD is commonly based on questionnaires, including the Gastrointestinal Symptom Rating Scale (GSRS) [6] and Frequency Scale for Symptoms of GERD (FSSG) [7].

Dyspepsia-related symptoms often coexist with those of GERD, although they are two distinct disorders. Functional dyspepsia (FD) is defined as epigastric pain or discomfort that persists for at least three months in patients without predominant heartburn or regurgitation without organic causes [2]. The Rome IV criteria continue to divide FD into postprandial distress syndrome (PDS), characterized by meal-related symptoms, and epigastric pain syndrome (EPS), typified by pain and burning [2].

### 1.2. Epidemiology

The estimated global prevalence of GERD is between 8% and 33% for all age groups and both genders. The prevalence also varies substantially between countries, most affecting populations in Western countries, including the Americas, Europe, Australia–New Zealand, and the Middle East (10% to 30%). It is less common in East Asia (<10%), and no data are available for Africa [1,8].

At least 10% to 20% of the US population reports weekly esophageal symptoms regarding heartburn and/or regurgitation [8]. In addition, GERD is one of the main healthcare issues in North America and Europe, placing a significant economic burden on society. For example, in the US, GERD is a common reason for consultation in primary and secondary care, with estimated costs that exceed 10 billion USD per year [8,9].

### 1.3. Pathophysiology

GERD is a chronic relapsing condition that occurs when gastric refluxate from the stomach, consisting of acid, pepsin, duodenal content and pancreatic enzymes, induces troublesome symptoms and/or complications in patients. The mechanisms of the pathogenesis of GERD include but are not limited to motor dysfunctions, hiatal hernia, and impaired mucosal resistance [5,10].

### 1.4. Management and Treatment

Stepwise management of GERD comprises lifestyle and dietary changes, followed by medical treatments that suppress intragastric acid secretion, including proton pomp inhibitors (PPIs). Lifestyle and nutritional changes can focus on modifications to diet, such as reducing the portion size per meal, consuming low-fat and low-protein foods, avoiding dietary and lifestyle triggers (e.g., nicotine, caffeine, and alcohol), and allergens (e.g., dairy and gluten). In addition, dietary supplements have been suggested to alleviate the severity and frequency of symptoms. Beneficial supplements include deglycerized licorice, glutamine, digestive enzymes, magnesium, and probiotics [9].

### 1.5. Probiotics and GERD

Probiotics are defined as ‘live microorganisms that, when administered in adequate amounts, confer a health benefit on the host’ [11]. Probiotics are available in a variety of forms, such as powders, capsules, foods, and infant formula [12]. The administration of probiotics has been recognized to benefit the health of the gut by improving bowel functions and abdominal symptoms [13,14]. The mechanisms of probiotics have been suggested to involve a wide range of activities, including direct interactions with the gut luminal microbiota, metabolic effects that result from enzymatic activities, effects on barrier function, and crosstalk with the central nervous system and enteric immunity [11,12,14]. Notwithstanding this, there is a lack of a thorough mechanistic understanding of probiotics’ functionality in general [15]; this is also the case for GERD.

The clinical implications of probiotics in gut health have been studied extensively in various clinical trials [14]. Although their ingestion does not appear to influence gastrointestinal microbiota in healthy adults [16], the consumption of probiotics during dysbiosis can promote gastrointestinal homeostasis and stimulate the growth of beneficial indigenous gut microbes [17]. Further, prior consumption of probiotics can reduce the risk of dysbiosis during conditions that challenge the composition of the intestinal microbiota, such as antibiotic use [18]. In general, supplementation with probiotics is related to benefits in the management of various lower-GI tract conditions, as documented in a recent systematic review of 70 randomized clinical trials that were published between 2012 and 2017 [14]. Highly supportive evidence of improvements in overall GI symptoms and abdominal pain in irritable bowel syndrome (IBS), and reductions in the risk of antibiotic-associated diarrhea and side effects that are associated with *Helicobacter pylori* (*H. pylori*) eradication therapy, were noted. Moderate evidence of improved bowel movements and bloating and distention in IBS was observed [14].

### 1.6. Aim of the Study

Most gastrointestinal benefits of probiotics have targeted the lower digestive tract, and limited data regarding the upper digestive tract have been reported, particularly for GERD. However, some of the biological events associated with GERD, such as changes in barrier function and immune response [19], are typically affected by probiotics; it can thus be hypothesized that they also play a role here [11]. Further, *Lactobacillus johnsonii* No. 1088 has been shown to reduce gastric acid production in an animal model [20]. *Bifidobacterium bifidum* YIT 10347 was shown to adhere to stomach cells and promote production of mucin, improving the physical gastric barrier to acidic stomach content [21]. Moreover, *B. bifidum* YIT 10347 regulates NF–kB signaling in more severe diseases, such as *H. pylori*-associated gastritis. The synergistic effects of these mechanisms can alleviate visceral hypersensitivity and improve the interactions between luminal contents and host esophageal epithelium in GERD. *Lactobacillus gasseri* LG21 has been shown to increase pepsinogen (PGI), which may contribute to improved digestion and shortened gastric residence time [22]. This indicates that there are potential mechanisms for the benefits of probiotics in GERD.

The aim of this systematic review was to examine the efficacy of probiotics in alleviating the symptoms, incidence, and severity of GERD in the general adult population, as this has not been done before. Although probiotics may be beneficial for patients receiving PPI treatment, we focused on studies with non-medicated subjects [23]. Due to the difficulty in distinguishing GERD from heartburn, regurgitation, and dyspepsia, this review will not differentiate between them.

## 2. Method

This systematic review was performed according to the Preferred Reporting Items for Systematic Reviews and Meta-Analyses (PRISMA) guidelines.

### 2.1. Literature Search

An advanced-mode electronic search was performed in the PubMed and Web of Science databases for prospective controlled studies using the terms “GERD OR dyspepsia OR heartburn OR regurgitation AND probiotic” in all age groups. We also performed focused searches of the Directory of Open Access Journals, Google Scholar, and reference lists of the included papers and applicable meta-analyses. The final search was performed in June 2019; eligible articles up to that date were considered for inclusion.

### 2.2. Study Selection and Data Extraction

**Figure 1:**
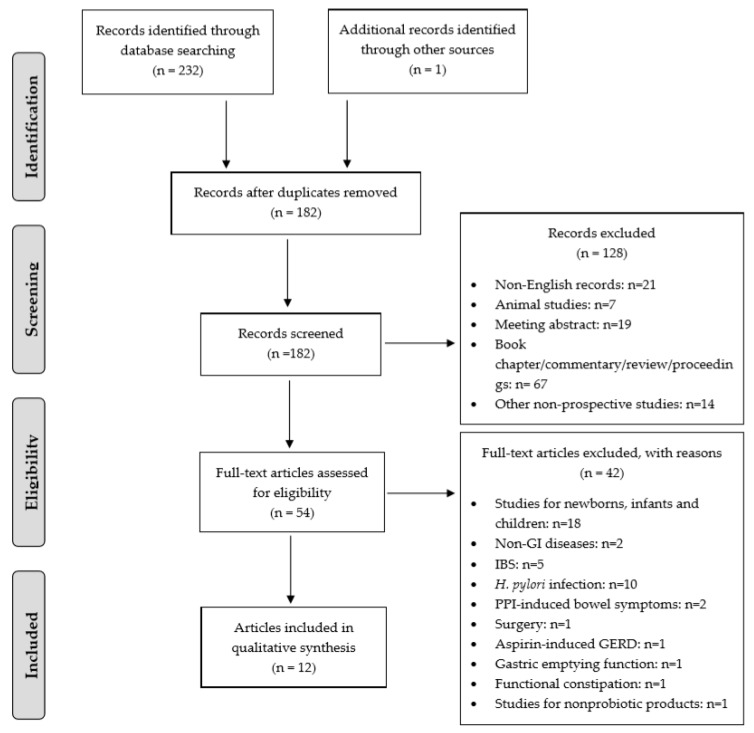
Preferred Reporting Items for Systematic Reviews and Meta-Analyses study flow diagram. Abbreviations: non-gastrointestinal (non-GI), irritable bowel syndrome (IBS), proton pump inhibitor (PPI), gastroesophageal reflux disease (GERD).

Two independent reviewers (J.C., A.C.O.) identified studies for inclusion and analyzed the selected articles. Discrepancies were resolved by discussion. The process of the study selection is illustrated in Figure 1. Titles and abstracts were first reviewed to exclude manuscripts that were published in non-English-language journals, systematic and literature reviews, commentaries, meeting abstracts, letters, case reports, animal studies, and clearly irrelevant studies. The remaining full-text articles were assessed for eligibility, based on the research questions. Data on subject characteristics (gender, age), study characteristics (study design, randomization, blinding, sample size, probiotic delivery vehicle, probiotics species/strain, daily probiotic dose, intervention duration), and clinical outcomes were recorded. The included clinical trials were scored using the Jadad scale [24] (Table S1).

## 3. Results

The database searches retrieved 232 titles and abstracts, and a manual search of relevant bibliographies identified one additional record. After the removal of duplicates, 182 unique titles remained. These titles and abstracts were screened for eligibility; 128 records were excluded, and 54 full-text articles were reviewed. In the analysis, 12 articles were included. One of the articles reported two interventions [25], and one article reported two probiotic study arms and one shared placebo arm [26]. Thus, the analysis ultimately included 14 comparisons. A flow diagram of the identification and selection of studies is shown in Figure 1.

A total of 951 subjects (mean: 68, range: 8–249/comparison) were analyzed in the 14 comparisons that were published in the 12 included articles. The subjects were healthy adults, including elderly persons. In most studies, both genders were evenly distributed in the analyzed population (Table 1). Daily probiotic doses ranged from 0.05 × 10^9^ to 46 × 10^9^ colony-forming units (CFU) (mean 5.8 × 10^9^ CFU). Treatment durations ranged from 1 to 12 weeks (mean six weeks) (Table 1).

A total of eight probiotic or synbiotic products were studied, containing between one and six strains (Table 1). Ten were single-strain products—*L. gasseri* LG21, *B. bifidum* YIT 10347, *Bifidobacterium animalis* subsp. *lactis* HN019, and *Lactobacillus reuteri* DSM 17938—whereas the four remaining products were multi-strain products, containing various strains in species of *B. bifidum*, *B. lactis*, *Bifidobacterium longum* subsp. *longum*, *Lactobacillus casei*, *Lactobacillus plantarum*, *Lactobacillus rhamnosus*, and *Lactobacillus acidophilus*. Four study products also contained other ingredients, such as antioxidants and prebiotics. In the included studies, the probiotics were administered in various formats: fermented dairy (seven comparisons), pill-like (four comparisons), powder (two comparisons), and olive oil (one comparison) (Table 1).

Of the 13 included studies, six were randomized and seven performed blinding of the patients; various study designs were used, including parallel groups (six studies), before–after comparisons (five studies), and crossover designs (two studies) (Table 2). After qualitative rating of the study design, per the Jadad scale, five randomized controlled trials (RCTs) with a parallel-group design were defined as high-quality, two RCTs with a parallel-group or crossover design were medium-quality, and the six remaining studies were low-quality (Table 2). Although it is not part of the Jadad score, reporting on compliance is an important marker of quality. Nearly half of the comparisons (*n* = 6) did not report compliance with the product (Table 3)

As shown in Table 3, of the 14 comparisons, 3-Gomi et al. (2015) study A and Waller et al. (2011) studies A and B reported significantly reduced (acid) regurgitation, and three comparisons [30,31,32] did not report any improvement. With regards to reflux or heartburn, two comparisons noted a significant improvement [22,27]. Trends for improvement were observed by [31] with the modified GSRS and by [21] with the original GSRS, but not with the FSSG questionnaire. Five comparisons reported no improvement: [25] Studies A and B and [21,30,32]. The remaining comparisons did not assess or report reflux syndrome symptoms.

Dyspepsia-related symptoms improved or declined in five comparisons: Gomi et al. (2015) study A and [21,28,29,30]. One study reported increased symptoms for dyspepsia [22], and three studies found no difference [31,32,33].

Five comparisons recorded a significant reduction in pain (abdominal or epigastric): Gomi et al. (2015) Study A, Waller et al. (2011) Studies A and B, and [27,28,29]. Two studies saw trends in reduced pain in the upper-GI region [30,31], and one reported no effect [32]. Three comparisons reported significantly less nausea: Waller et al. (2011) Studies A and B and [28]. Improvements in gas-related upper-GI symptom severity were observed in four comparisons: Gomi et al. (2015) Study A, Ianiro et al. (2013), and Waller et al. (2011) Studies A and B. One study noted an improvement in the prevalence of flatus [31].

Overall, of the 13 selected studies, 11 comparisons (79%) reported probiotic benefits on the symptoms of GERD, whereas no benefit was seen in the three remaining comparisons [32,33,34]. Of the 11 former comparisons, five (45%) reported benefits for reflux symptoms, versus five (45%) for dyspepsia symptoms and nine (81%) for other upper-GI symptoms, such as nausea, abdominal pain, and gas-related symptoms (belching, gurgling, burping). Of the five high-quality RCTs, two comparisons in Waller et al. (2011) showed efficacy (40%) with regard to reflux symptoms and other upper-GI symptoms, primarily gas-related symptoms; one study (20%) [30] noted improvements in dyspepsia-related symptoms.

Three comparisons reported benefits for regurgitation, using frequency score as the endpoint, in which two single-strain probiotics were used: *B. bifidum* YIT 10347 in Study A in [25] and *B. lactis* HN019 for two comparisons in [26]. Gomi et al. (2015) in Study A reported a lower frequency of regurgitation. Waller et al. (2011) observed similar efficacy in reducing the frequency score for regurgitation at a high dose (17.2 × 10^9^ CFU) by 12.6, and by 9.0 at the lower dose (1.8 × 10^9^ CFU) [30].

In this review, four comparisons were performed with *B. bifidum* YIT 10347 with the same intervention regimen, but only one showed an effect on both regurgitation and dyspepsia—Study A in [25]—one saw improvements in acid-related dyspepsia [21], and two reported positive effects on gas-related symptoms [25,31].

Most studies (*n* = 9) recorded adverse events (AEs) (Table 3), but none were associated with the probiotic intervention. No serious adverse events were reported.

## 4. Discussion

In this systematic review, 13 prospective clinical studies, comprising 14 comparisons, were reviewed to determine the potential of probiotics to alleviate upper-GI symptoms in GERD in the general adult population. The mechanism of action of probiotics has focused primarily on the lower digestive tract, and the activities of probiotics in the upper-GI tract remain largely unknown [15].

Nevertheless, probiotics of the genera *Lactobacillus* and *Bifidobacterium* are associated with modulations in the immune response and antagonistic activity toward potential pathogens through the production of short-chain fatty acids, such as lactic acid. Further, probiotics accelerate gastric emptying by interacting with stomach mucosal receptors, which are suspected of triggering transient lower esophageal sphincter relaxation, one of the pathophysiological mechanisms of GERD [35]. In addition, probiotics can be beneficial for small intestinal bacterial overgrowth, interfering with immunity or intestinal motility under various conditions [36]. These properties might be relevant to their effects in GERD, as discussed here.

### 4.1. Clinical Efficacy and Potential Mechanisms

A majority (79%) of the included comparisons reported probiotic benefits on the symptoms of GERD, such as regurgitation, heartburn, dyspepsia, nausea, abdominal pain, and gas-related symptoms (belching, gurgling, burping). However, the heterogeneity in the outcomes made it impossible to perform a meta-analysis.

Probiotics have positive effects on reflux with regards to the presence of episodes [27] and frequency scores [22]. The presence of reflux episodes fell significantly by 40% in 20 pregnant women [27]. To our knowledge, de Milliano et al. (2012) is the first trial to supplement with multi-strain probiotics, and reported benefits for reflux, particularly in constipated pregnant women. The product in this study contained six probiotic strains from six species, including *Bifibacterium* and *Lactobacillus*, providing efficacy for a wide range of upper- and lower-GI symptoms, such as abdominal pain and constipation [27].

Based on the FSSG, the frequency scores for reflux declined significantly from 6.2 to 4.8 on supplementation with *L. gasseri* LG21 for 12 weeks [22]. Notably, in the same study, pepsinogen (PGI) level was the only stomach-related biomarker that had a significant negative correlation with the reflux symptom score, after the effects of gender and age were adjusted [22]. PGI was suspected to be involved in the occurrence of symptoms; thus, a higher PGI level indicates accelerated protein digestion in the stomach. This explanation is one basis for the inverse relationship between increased PGI levels and reduced reflux symptoms, particularly in the presence of increased dysmotility-like dyspepsia, from 3.5 to 4.0 on the FSSG [22].

In addition to its involvement in protein digestion, other underlying mechanisms of *L. gasseri* LG21 in reflux and dyspepsia were examined in two cohorts [29,30]. In these two studies, no improvement in regurgitation or heartburn was seen, whereas both reported reduced/lower dyspepsia—i.e., reduced postprandial distress—regardless of the experimental design (self-controlled or placebo-controlled). Moreover, Nakae et al. (2016) observed increased gastric emptying, as evidenced by the increased gastric fluid volume and suppressed gastric acid secretion, based on a higher pH value after treatment. Although Ohtsu et al. (2017) focused only on symptomology, to better differentiate PDS from EPS, they administered questionnaires other than the FSSG in [22]. Notably, compared with the placebo, postprandial distress syndrome scores declined significantly with the *L. gasseri* LG21 intervention (37.5% vs. 17.8%), whereas only a trend of improvement for epigastric burning, with no improvement in epigastric pain, was reported, indicating that *L. gasseri* LG21 has greater beneficial effects on PDS symptoms than EPS [30]. These findings strongly suggest that the underlying mechanisms of *L. gasseri* LG21 for improving FD-associated reflux are linked to postprandial effects, involving improved protein indigestion and increased gastric emptying.

Little is known about gastric microbiota and its function in the pathogenesis of GERD and FD [29]. In Nakea et al. (2016), FD patients had a clearly different bacterial community compared with healthy controls, in terms of overall community structure and bacterial taxonomic abundance. *Prevotella* spp. is the predominant taxa inhabiting the stomach [29]. In this study, the relative abundance of *Prevotella* spp. was lower in FD patients versus healthy controls and significantly negatively correlated with the severity of postprandial distress symptoms scores, implicating *Prevotella* spp. in the occurrence of FD symptoms.

*H. pylori* infection is associated with symptoms of GERD. However, its eradication is not always associated with an improvement in symptoms [37]. In view of this, it is unfortunate that the studies have not evaluated the presence or absence of *H. pylori*. Although probiotics do not eradicate *H. pylori*, they have been shown to reduce its activity [38]. Information on the carriage of *H. pylori* could thus have given information on some of the differences in efficacy.

### 4.2. Effects of Product Format on Efficacy

Limited evidence is available to compare efficacy between probiotic strains, due to the small number of available trials. However, all three included *L. gasseri* LG21 studies reported positive effects on various symptoms of GERD. Also, for *B. bifidum* YIT 10347, four intervention trials indicated positive changes in FSSG and GSRS scores. For other strains, too few studies were available to draw overall conclusions. Very diverse product formats were applied in the selected studies, ranging from fermented foods to various dietary supplement formats.

### 4.3. Safety

Although adverse events were reported in three studies [30,31,32], none differed significantly between probiotic and placebo groups. Moreover, the adverse events were assumed not to be product-related effects.

### 4.4. Study Quality

A potential source of bias for the experimental design was assessed in Table 2. As demonstrated in [39], the level of evidence for trials with different experimental designs could be classified as follows (in descending order): high-quality RCTs, low-quality RCTs, prospective cohort studies, and others. All six RCTs were randomized and double-blinded with an identical appearance of placebo and probiotic-containing products. However, especially in food, it might be challenging to manufacture a placebo that is indistinguishable from the probiotic product.

Half of the studies implemented random sequence generation by computer-based randomization programs, block approach, or tables. However, the allocation concealment was not clear in these studies. Adequate allocation concealment is important for decreasing the risk of selection bias in clinical trials [40]. The odds ratio for the estimated effects of inadequate allocation concealment on treatment can be as high as 41%, having been the only risk of bias until 2008, as reported by RevMan, a program that was used for Cochrane Reviews [41]. These findings suggest that inadequate allocation concealment is the leading cause of bias. Two RCTs also had an unclear risk of bias due to the lack of a clear description of the reasons for withdrawal or dropouts.

Although it is not part of the Jadad score, reporting on compliance is an important quality marker. Nearly half of the comparisons did not report compliance with the product, including two studies that were judged to be high-quality. Future systematic reviews and meta-analyses should take product compliance into account in their quality evaluation.

## 5. Conclusions

Most studies reported positive outcomes for probiotics regarding the symptoms of GERD. However, there was substantial heterogeneity in the outcomes and symptoms. Thus, although the results are encouraging, it is difficult to draw any general conclusions on the effects of probiotics. The heterogeneity in endpoints also made it impossible to quantitively evaluate the results. Further, the quality of the studies is concerning—only 5 of 14 studies were good quality. Nevertheless, despite the diversity in the studied product formats, populations, and experimental designs, the efficacy of the probiotic treatment does not appear to be influenced by the study quality.

Properly designed, randomized, double-blind, placebo-controlled studies with a sufficient number of participants and well-defined endpoints are needed. Studies with a longer duration should also be considered, with an intermediate analysis of the endpoints—for example, through questionnaires—to determine the period in which the benefits can be expected and whether they are long-lasting.

## Figures and Tables

**Table 1:** Study Characteristics.

Study	Number of Subjects (Probiotic: Control)	Female (%)	Age (mean ± SD, Range) [I]	Delivery Vehicle	Probiotic Strain(s) [II]	Dose (10^9^ CFU/day)	Intervention Duration (wk)
[27]	20 (20:0)	100	29.5 ± 5.3 ^#^	NR (sachet/stick)	*B. bifidum* W23, *B. lactis* W52, *B. longum* W108, *L. casei* W79, *L. plantarum* W62, *L. rhamnosus* W71 and o FOS, inulin	4	4
[28]	8 (8:8)	NR	NR but suspected to be adults	Olive oil	*L. reuteri* *, *L. rhamnosus GG* *, *Saccharomyces boulardii* *, and vitamin B6 hydrochloride Q10 coenzyme	46	1
[29]	44 (44:0)	50%	42.5 (34.5–50.3) ^##^	Yogurt	*L. gasseri* LG21	1	12
[30]	106 (54:52)	75%	42.8 ± 9.0	Yogurt	*L. gasseri* LG21	>1	12
[22]	24 (24:0)	33%	68.6 ± 9.7	Yogurt	*L. gasseri* LG21	1	12
[25] A [III]	149 (149:149)	48%	50.6 ± 7.4 (33–84)	Fermented milk	*B. bifidum* YIT 10347	1	2
[25] B [III]	27 (27:27)	13%	35.3 ± 11.3 (21–58)	Fermented milk	*B. bifidum* YIT 10347	1	2
[31]	79 (39:40)	52%	Probiotic: 41.1 ± 10.1 Placebo: 41.6 ± 9.9	Fermented milk	*B. bifidum* YIT 10347	>3	4
[21]	37 (37:0)	51%	52.6 ± 17.5 (12–78)	Fermented milk	*B. bifidum* YIT 10347	>1	2
[26] A [IV]	54 (26:28)	62%	Probiotic: 44 ± 11 Placebo: 45 ± 11	Capsule	*B. lactis* HN019	1.8	2
[26] B [IV]	61 (33:28)	61%	Probiotic: 43 ± 12 Placebo: 45 ± 11	Capsule	*B. lactis* HN019	17.2	2
[32]	249 (125:124)	57%	Probiotic: 72.6 ± 5.8 Placebo: 72 ± 5.6	Stick	*L. reuteri* DSM 1793 and GOS	0.1	12
[33]	36 (18:18)	56%	NR (24–45)	Tablet	*L. acidophilus* La5, *B. lactis* Bb-12, *Lactobacillus bulgaricus* *, *Lactobacilus paracasei* *, *Streptococcus thermophilus* *, and FOS	2.4	6
[34]	24 (12:12)	75%	Probiotic: 41.1 ± 12:5 Placebo: 41.5 ± 15.8	Caplet	*L. acidophilus* *, *B. bifidum* *, *Bacillus subtilis* *, *L. bulgaricus* *, *L. lactis* *, *Bacillus licheniformis* *	0.05	12

Abbreviations: year (yr), standard deviation (SD), colony-forming units (CFU), week (wk), not recorded (NR), galacto-oligosaccharides (GOSs), fructo-oligosaccharides (FOSs), Deutsche Sammlung von Mikroorganismen (German Collection of Microorganisms, DSM). [I] Ages are expressed in years. ^#^ median ± SD. ^##^ median (interquartile range). [II] * Strain unspecified. [III] A and B represent two trials, termed Trials 1 and 2, in [25]. [IV] A and B represent low-dose and high-dose treatments in the same trial in [26].

**Table 2:** Study Design and Quality Rating.

Study	Randomization	Blinding	Design	Jadad Score [I]						Qualitative Rating [II]	IP Compliance
				(1)	(2)	(3)	(4)	(5)	Total		
[27]	No	No	Before-after	0	0	0	0	1	1	Low	100%
[28]	No	No	Crossover	0	0	0	0	1	1	Low	NR
[29]	No	No	Before-after	0	0	0	0	0	0	Low	NR
[30]	Yes	Yes	Parallel Group	1	0	1	1	1	4	High	NR
[22]	No	No	Before-after	0	0	0	0	1	1	Low	>90%
[25] A [IV]	No	No	Before-after	0	0	0	0	1	1	Low	>95%
[25] B [IV]	NR	Yes	Crossover	0	0	1	1	1	3	Medium	>95%
[31]	Yes	Yes	Parallel Group	1	0	1	1	1	4	High	>95%
[21]	No	No	Before-after	0	0	0	0	1	1	Low	NR
[26] A [V]	Yes	Yes	Parallel Group	1	1	1	1	0	4	High	100%
[26] B [V]	Yes	Yes	Parallel Group	1	1	1	1	0	4	High	100%
[32]	Yes	Yes	Parallel Group	1	1	1	1	1	5	High	NR
[33]	Yes	Yes	Parallel Group	1	0	1	1	0	3	Medium	NR
[34]	Yes	Yes	Parallel Group	1	1	1	1	1	5	High	>75%

Abbreviations: not reported (NR). [I] Points were rated for each item according to Table S1. [II] Total Jadad scores were classified into three categories: high- (4,5), medium- (3), and low-quality (0,1,2). [III] Each column corresponds to one type of upper-GI symptom, as presented in Table 3: (1) reflux symptoms, (2) dyspepsia-related symptoms, and (3) others. [IV] A and B represent two trials: Trial 1 and Trial 2 in [25]. [V] A and B represent low-dose and high-dose treatments in the same trial in [26].

**Table 3:** Study Design and Clinical Outcomes.

Study	Population	Inclusion Criteria	Side Effects/Adverse Events	Clinical Outcomes		
				Reflux Symptoms (Regurgitation/Acid Reflux/Heartburn)	Dyspepsia-Related Symptoms	Other upper-GI Symptoms
[27]	Pregnant Woman	Rome III for Functional Constipation	None	Reflux episode presence reduced significantly by 40%	NA	Episodes of abdominal pain reduced significantly by 40%
[28]	Adult	Rome III for Functional Dyspepsia	NR	NA	Significantly reduced postprandial gastric distension and postprandial fullness compared with placebo	Compared with placebo, significantly reduced nausea and pain/discomfort in abdominal upper quadrants and relief of belching
[29]	Adult	Rome III for Functional Dyspepsia	NR	NR	Significantly reduced postprandial distress by 7.7 points in FSSG	Significantly reduced epigastric pain by 8 points in FSSG
[30]	Adult	Rome III for Functional Dyspepsia	No difference in adverse events (AEs) between probiotic (*n* = 2) and placebo (*n* = 5)	No significant reduction in regurgitation or heartburn at endpoint (week 12), but at Week 8, a significant decrease in both symptoms was observed	Significantly reduced overall FD symptom score compared with placebo (35.2 vs. 17.3%). Postprandial distress syndrome score was significantly lower versus placebo (37.5 vs. 17.8%).	A trend for the improvement in epigastric burning (*p* = 0.086).
[22]	Adult + Elderly	Patient’s medical history, upper-GI endoscopy and FSSG	NR	Frequency score of reflux reduced significantly, from 6.2 to 4.8	Significantly increased dysmotility-like dyspepsia, from 3.5 to 4.0 on the FSSG	Overall FSSG score reduced significantly from 10.8 to 8.4.
[25] A [I]	Adult	Modified GSRS for gastric symptoms	None	Significantly reduced acid regurgitation, no effect on reflux	Significantly lower individual symptom scores for stomach heaviness	Compared with baseline, significantly fewer gastric symptoms by 0.8 and 1.1 and reduced overall gastric symptom score by 0.9 and 1.2 after 1 and 2 weeks, respectively. Significantly reduced individual symptom scores in burp, no appetite, and repeated abdominal pain or discomfort.
[25] B [I]	Adult	Modified FSSG for gastric symptoms	None	No effect on regurgitation or reflux	NR	The modified F-scale score was significantly reduced by 1.0 and 1.1 after 1 and 2 weeks compared with baseline, no comparison with placebo.
[31]	Adult	Modified FSSG but not Rome IV for Functional Dyspepsia	No difference between probiotic (*n* = 7) and placebo (*n* = 12).	Compared with placebo, no difference in modified FSSG for reflux syndrome. No difference in acid regurgitation but a trend for improved heartburn (−0.90 vs. −0.38) in GSRS.	On the modified FSSG, no difference in acid-related dyspepsia but trend toward postprandial discomfort (−0.56 vs. −0.33)	On the modified FSSG, a trend for improvement in burping (−0.62 vs. −0.38) and postprandial epigastric pain (−0.38 vs. −0.08). On the GSRS, trend for improved upper-GI symptoms (−0.72 vs. −0.45). No difference in severity but significantly lower prevalence of gas-related symptoms (flatus).
[21]	Adult	Functional gastrointestinal disorder by physician	AEs (*n* = 2) for intestinal gas and bloating	No difference for reflux symptoms on the FSSG, but a trend in GSRS (*p* = 0.06).	Significantly decreased indigestion syndrome scores on the GSRS and acid-related dyspepsia on the FSSG.	Significantly decreased overall GSRS and FSSG scores.
[26] A [II]	Adult	Self-reported constipation	None	Significantly lower frequency score for regurgitation by 11.3 vs. 2.3 (placebo)	NA	Significantly lower frequency score for nausea, abdominal pain, gurgling.
[26] B [II]	Adult	Self-reported constipation	None	Significantly lower frequency score for regurgitation by 14.9 vs. 2.3 (placebo)	NA	Significantly lower frequency score for nausea, abdominal pain, gurgling, and vomiting.
[32]	Elderly	GI discomfort defined by a score of >2 in any domain on the GSRS	No difference of AEs between probiotic and placebo. Serious adverse events: none	No effect on regurgitation or reflux	No effects on indigestion/dyspepsia (−0.14 vs. −0.13).	No effects on abdominal pain (−0.08 vs. −0.09).
[33]	Adult	Rome II for dyspepsia, postprandial bloating, constipation, flatulence	None	NA	No difference in dyspepsia	NA
[34]	Adult	Rome II for functional bowel disorder	None	NR	NR	A trend toward improved general GI symptoms (lower and upper GI), reduced by 18.9% vs. 8.8% with placebo.

Abbreviations: gastrointestinal (GI), not assessed (NA), not reported (NR), Frequency Scale for the Symptoms of Gastroesophageal Reflux Disease (FSSG), Gastrointestinal Symptoms Rating Scale (GSRS), adverse events (AEs). [I] A and B represent two trials: Trial 1 and Trial 2 in [25]. [II] A and B represent low-dose and high-dose treatments in the same trial in [26].

## Supplementary Materials

The following are available online at https://www.mdpi.com/2072-6643/12/1/132/s1, Table S1: Jadad scale rating items of controlled clinical trials [24].
